# Th1-Polarized, Dengue Virus-Activated Human Mast Cells Induce Endothelial Transcriptional Activation and Permeability

**DOI:** 10.3390/v12121379

**Published:** 2020-12-02

**Authors:** Ayesa Syenina, Wilfried A. A. Saron, Cyril J. Jagaraj, Siham Bibi, Michel Arock, Duane J. Gubler, Abhay P. S. Rathore, Soman N. Abraham, Ashley L. St. John

**Affiliations:** 1 Program in Emerging Infectious Diseases, Duke-National University of Singapore, Singapore 169857, Singapore; ayesa.syenina@duke-nus.edu.sg (A.S.); wilfried.saron@duke-nus.edu.sg (W.A.A.S.); cyril-jones.jagaraj@students.mq.edu.au (C.J.J.); duane.gubler@duke-nus.edu.sg (D.J.G.); soman.abraham@duke.edu (S.N.A.); 2 Laboratory of Molecular and Cellular Oncology, LBPA CNRS UMR8113, Ecole Normale Supérieure de Cachan, 94235 Cachan, France; syham_bibi@hotmail.com (S.B.); michel.arock@ens-cachan.fr (M.A.); 3 Laboratory of Hematology, Pitié-Salpêtrière Hospital, Pierre et Marie Curie University, 75005 Paris, France; 4 Pathology Department, Duke University Medical Center, Durham, NC 27710, USA; abhay.rathore@duke.edu; 5 Immunology Department, Duke University Medical Center, Durham, NC 27710, USA; 6 Molecular Genetics and Microbiology Departments, Duke University Medical Center, Durham, NC 27710, USA; 7 Department of Microbiology and Immunology, National University of Singapore, Singapore 117545, Singapore; 8 SingHealth Duke-NUS Global Health Institute, Singapore 169857, Singapore

**Keywords:** dengue, mast cell, human, endothelial permeability, chymase

## Abstract

Dengue virus (DENV), an arbovirus, strongly activates mast cells (MCs), which are key immune cells for pathogen immune surveillance. In animal models, MCs promote clearance of local peripheral DENV infections but, conversely, also promote pathological vascular leakage when widely activated during systemic DENV infection. Since DENV is a human pathogen, we sought to ascertain whether a similar phenomenon could occur in humans by characterizing the products released by human MCs (huMCs) upon direct (antibody-independent) DENV exposure, using the phenotypically mature huMC line, ROSA. DENV did not productively infect huMCs but prompted huMC release of proteases and eicosanoids and induced a Th1-polarized transcriptional profile. In co-culture and trans-well systems, huMC products activated human microvascular endothelial cells, involving transcription of vasoactive mediators and increased monolayer permeability. This permeability was blocked by MC-stabilizing drugs, or limited by drugs targeting certain MC products. Thus, MC stabilizers are a viable strategy to limit MC-promoted vascular leakage during DENV infection in humans.

## 1. Introduction

Mast cells (MCs) are granulated cells found at the interface between the host and environment, such as in the skin and mucosa [1,2]. Some are distributed at regular intervals throughout connective tissues, where a portion of these can be found in close proximity to blood and lymph vessels [2,3]. As key innate immune cells, they are involved in the earliest phase of host defense due to their capacity to detect pathogens and rapidly degranulate and/or begin production of pro-inflammatory lipid and protein mediators, the combination of which are frequently pathogen- or stimulus-specific. Products that MCs secrete during the acute inflammatory response include preformed mediators and de novo synthesized products, such as histamine, MC-specific proteases, eiocosanoids (e.g., leukotrienes and prostaglandins), cytokines and chemokines [2]. Many of these products are known to contribute to endothelial activation, regulate the coagulation cascade, promote vascularization and/or increase vascular permeability [4,5].

While MCs have been mostly studied in the context of allergic responses, they are also implicated in host defense against bacterial, helminth, and, more recently, viral infections [2,5,6,7,8,9]. A key viral pathogen of interest is dengue virus (DENV), since this arboviral pathogen is injected into the skin (where MCs are highly abundant) by mosquitos. DENV is a positive-sense RNA-enveloped virus from the Flavivirus genus that is thought to replicate primarily in monocytes, dendritic cells, and similar phagocytic cell types in vivo [10,11]. In humans, DENV disease is characterized by a febrile illness involving signs of vascular leakage that range from mild to severe clinical manifestations; when mild, referred to as dengue fever (DF) and when severe, termed dengue hemorrhagic fever (DHF) [12]. More recently, the WHO has updated the dengue diagnosis guidelines to include dengue with and without warning signs and severe dengue. While warning signs include abdominal pain, persistent vomiting, clinical fluid accumulation, mucosal bleeding, lethargy or restlessness, liver enlargement, or increased hematocrit concurrent with rapidly decreasing platelet counts, severe dengue involves severe plasma leakage leading to shock or respiratory distress, severe bleeding, and severe organ involvement [13]. MCs have been shown to degranulate in response to the structural components of DENV and have been implicated in DENV-induced vascular leakage in a mouse model of DENV [7,9,14]. In this rodent model, MC-stabilizing drugs were also shown to block DENV-induced vascular leakage in vivo and reverse pro-inflammatory transcriptional signatures in tissues [14,15]. Furthermore, MC degranulation to DENV can be enhanced in the presence of DENV-specific antibodies due to cross-linking of immunoglobulin Fcγ receptors in vitro and in vivo [16].

Most previous studies examining the responses of MCs to DENV have predominantly investigated the responses of rodent MCs to DENV, although DENV is a human pathogen. In support of the fact that human MCs (huMCs) respond to DENV, we previously reported that chymase, a protease produced specifically by MCs, is elevated in the serum of DENV patients, and serum levels are correlated with severity of disease [14,17]. More recently, we showed that MC-specific proteases, tryptase and chymase were functionally consequential for DENV-induced vascular leak and shock in mice and correlated with grades of DHF in humans [18]. These studies confirmed that MCs are activated by DENV in humans, and emphasize the importance of understanding which huMC products are released in response to DENV and how those products might influence other cell types. However, a major limitation to studying huMC responses to pathogens has been the lack of cell lines that recapitulate the features of mature peripheral tissue-resident MCs. Several cell lines that have been described as MCs are hypogranulated, such as the HMC-1 cell line, which also often expresses a low level of FcεR1 in culture [19]. We have previously reported that another huMC line, tumor-derived LAD2, degranulates in response to direct stimulation by DENV (i.e., DENV-specific antibody is not required for MC activation and degranulation) [7]; however, LAD2 cells also have limitations in that they are extremely difficult to propagate in culture and they replicate very slowly, limiting the feasibility of further characterization of their responses to DENV. Ku-812 have been described to approximate MCs, but these cells are also phenotypically inconsistent with mature MCs [20]. The consensus is that this human leukemia cell line more closely resembles immature pre-basophilic cells [20]. However, Ku-812 cells have also been used in studies showing that they can become substantially infected in the presence of DENV-specific antibody, resulting in production of infection-related cytokines such as TNF [21]. These limitations of cell culture systems impeded investigations seeking to understand how mature huMCs, which are only found resident in tissues, would respond to DENV.

In this study, we used a recently developed huMC cell line, ROSA, to characterize the responses of mature huMCs to DENV. ROSA cells are highly granulated cells derived from human cord blood [22]. They are stem cell factor (SCF)-dependent and express the FcεR1 receptor [22]. We characterized the responses of ROSA cells to DENV in terms of degranulation, cytokine transcription and eiocosinoid mediator production. Our results showed that the vasoactive mediators released by huMCs promote the permeability of human microvascular endothelial cell (huMEC) monolayers and induce endothelial activation. Like mouse MCs [14,15,16], DENV-activated huMCs were also sensitive to drugs in the class of MC-stabilizers, implying the feasibility of using MC stabilizers to block DENV vascular leakage in humans.

## 2. Materials and Methods

### 2.1. Cell Lines, Virus Strains and Culture Conditions

The huMC line used in these studies was sub-cloned from the ROSA, a cord blood-derived MC line, which has a normal wild-type *KIT* gene [22]. For these experiments, ROSA cells were propagated in RPMI media containing 10% FCS (Gibco-Invitrogen, Thermo Fisher Scientific, Waltham, MA, USA), penicillin and streptomycin (100 µg/mL each, Invitrogen, Thermo Fisher Scientific) and 1% supernatant from CHO-KL cells (a cell line engineered to release recombinant SCF). The huMEC cell line HMVEC-d Ad-Dermal MV Endo Cells was maintained in EGM-2MV BulletKit media (both from Lonza, Walkersville, MD, USA). Hamster kidney cells, BHK21 and mouse monocyte/macrophage cells J774A.1 were also obtained from ATCC (Manassas, VI, USA) and maintained in RPMI and DMEM media (both obtained from Gibco-Invitrogen), respectively. The human clinical isolate of DENV2, strain Eden2, which was obtained from the Early Dengue Infection and Outcomes Study (Eden) [23], was maintained as previously described [7,14].

### 2.2. huMC Infections and Assessments of Activation

huMCs were incubated with DENV at an MOI of 1. To visualize huMC activation, cells were exposed to DENV for 1 h prior to cytospinning the cells onto glass slides, followed by Wright–Giemsa staining. Images were obtained by light microscopy at 100× magnification. To assess degranulation, β-hexosaminidase was measured in huMC supernatants and cell lysates after 1 h exposure to DENV-containing media, media alone, or 0.5 µg/mL ionomycin. Media from c6/36 cells used to propagate DENV was confirmed to not induce degranulation itself. Since the ROSA cell line is non-adherent, cells were pelleted by centrifugation prior to collecting supernatants. Cell pellets were then solubilized in 0.1% Triton X-100 (Bio-rad, Hercules, CA, USA). The enzyme activity was measured with *p*-nitrophenol-*N*-acetyl-β-d-glucosaminide in 0.1 M sodium citrate (pH 4.5) for 1 h at 37 °C. The reaction was stopped by the addition of 0.1 M carbonate buffer (pH 10.0). The release of the product 4-*p*-nitrophenol was detected by absorbance at 405 nm. Percentage degranulation was calculated by dividing the absorbance in the supernatant by the sum of absorbance in the supernatant and cell pellet. To measure products released by huMCs, supernatants were collected after exposure to DENV (or control media) for the designated time points and used to measure prostaglandins and leukotrienes by ELISA kits, following the manufacturer’s instructions (Prostaglandin ELISA kit ABIN773544 and Cysteinyl Leukotrienes ELISA kit ABIN627737, both from antibodies-online, Aachen, Germany). Transcriptional changes after DENV exposure were assessed using the Inflammatory Response & Autoimmunity PCR array (PAHS-077ZA, Qiagen, Germantown, MD, USA), and data were obtained and analyzed according to the manufacturer’s instructions. IL-12 transcriptional activation was evaluated using published primers [24].

### 2.3. Measurement of Endothelial Activation and Permeability

huMECs were grown in 3 µm inserts inside 24-well plates for 4–5 days to form a monolayer. Separately, 3 × 10^6^ huMCs were treated for 1 h with either DENV alone, DENV and MC-stabilizing drugs, or with media alone (untreated). For MC-stabilizing compounds, cromolyn or ketotifen (10 µM final concentration, Sigma-Aldrich, St. Louis, MO, USA) were incubated with huMCs during exposure to DENV. The leukotriene receptor antagonists (montelukast, 10 µM, Sigma) or protease inhibitors (chymostatin, 50 µM, Sigma-Aldrich) were added directly to the huMEC monolayer during exposure of the monolayers to the 500 µL of huMC supernatants. Monolayer permeability was measured using two parameters. First, trans-endothelial electrical resistance (TER) readings were obtained at baseline and 24 h after treatment with DENV-elicited MC supernatants or appropriate controls using the MilliCell-ERS system (Millipore-Sigma, St. Louis, MO, USA). Second, FITC-conjugated dextran diffusion across the monolayer was measured, by adding 10 mg/mL of FITC-dextran (Sigma-Aldrich) to inserts 24 h after treatment. At 48 h, supernatants were collected from the opposite side of the monolayer and the amount of FITC-dextran that diffused across the monolayer was quantitated by measuring the fluorescence intensity at 490 nm. For PCR arrays, huMECs were grown in 24-well plates overnight to 90–100% confluence. Separately, 3 × 10^6^ huMCs were treated with DENV, as described above, and huMC supernatants were collected after 1 h incubation and transferred onto huMEC cells. After incubation for 24 h, RNA was extracted from huMECs using the Trizol method (Life Technologies, Thermo Fisher Scientific). cDNA was synthesized using the RT2 First strand kit (Qiagen) and was then used for the Human Endothelial Cell Biology PCR Array (PAHS-015ZD-2, Qiagen), following the manufacturer’s instructions.

### 2.4. Immunofluorescence Assay

HuMECs were grown on coverslips (Warner Instruments, Holliston, MA, USA) inside 24-well plates for 2 d to form a monolayer. huMCs were treated with DENV ± MC-stabilizing drugs, as described above. Leukotriene antagonists or protease inhibitors were added to the huMEC monolayers, also as above, concurrently with the transfer of huMC ± DENV supernatants onto huMCs. Treated huMECs were incubated for 24 h, followed by fixing with paraformaldehyde. Coverslips were then washed with PBS and blocked using 0.1% saponin in 1% BSA in PBS (permeabilizing buffer). Primary antibodies against α-tubulin (GeneTex, Irvine, CA, USA) and ZO-1 (Invitrogen, Thermo Fisher Scientific, Waltham, MA, USA) were added to permeabilization buffer and incubated overnight followed by washing using permeabilizing buffer. Next, the secondary antibodies, anti-mouse-conjugated FITC (Jackson ImmunoResearch, West Grove, PA, USA) and anti-rabbit-conjugated AlexaFlour660 (Moleculer Probes) were added in permeabilizing buffer and incubated for 2–4 h. Finally, coverslips were mounted using Pro-Long Gold Anti-fade reagent containing DAPI (Invitrogen, Thermo Fisher Scientific). Cell images were obtained using the LSM710 Carl Zeiss Confocal Microscope (Carl Zeiss Microscopy Deutschland GmbH, Oberkochen, Germany) at 20× and 63× magnification using a channel series approach to diminish spectral overlap.

For immunostaining of HuMCs and J774A.1 cells for the detection of DENV, cells (1 × 10^6^ HuMCs, and 1 × 10^5^ J774A.1) were infected with DENV (MOI of 1 for huMCs and MOI of 0.5 for J774A.1 cells). Infected cells were washed with their respective maintenance media to remove excess virus from the cells followed by incubation of 48 h to allow virus replication to occur. At 48 h post-infection, cells were fixed using 80% acetone and blocked using 2% BSA in PBS for 2 h at room temperature. Cells were incubated with primary antibody clone 4G2 (ATCC, Manassas, VI, USA), against the DENV envelope protein, overnight at 4 °C. Cells were washed 3 times using PBS for 10 min each time at room temperature, followed by secondary antibody staining using anti-mouse-conjugated AlexaFlour555 (Invitrogen, Thermo Fisher Scientific) for 1 h at room temperature. After secondary antibody staining, cells were washed 2× using PBS and 2× using distilled water before incubation with Sytox Green nucleic acid stain (Invitrogen, Thermo Fisher Scientific) for 20 min. Finally, cells were washed 2× with distilled water and 2× with PBS before mounting using ProLong Gold antifade reagent (Invitrogen, Thermo Fisher Scientific). Confocal images of stained cells were obtained using a three-laser Nikon confocal scanning instrument (Nikon, Melville, NY, USA).

### 2.5. DENV Infection Kinetics in huMCs and Macrophage Cells

Cells (1 × 10^6^ HuMCs or 1 × 10^5^ J774A.1) were infected with DENV using an MOI of 1 and 0.5, respectively. J774A.1 cells were chosen to serve as a positive control since they are known to support DENV replication. Infected cells were washed once with their respective maintenance media to remove excess virus from the cells and incubated in maintenance media for various time points post-infection (6 h, 24 h, 48 h and 72 h). At indicated time points post-infection, cell supernatants were collected for quantification of infectious virus particles using a standard plaque assay [25].

## 3. Results

### 3.1. Release of Vasoactive Products by DENV-Activated Human MCs

**Figure 1:**
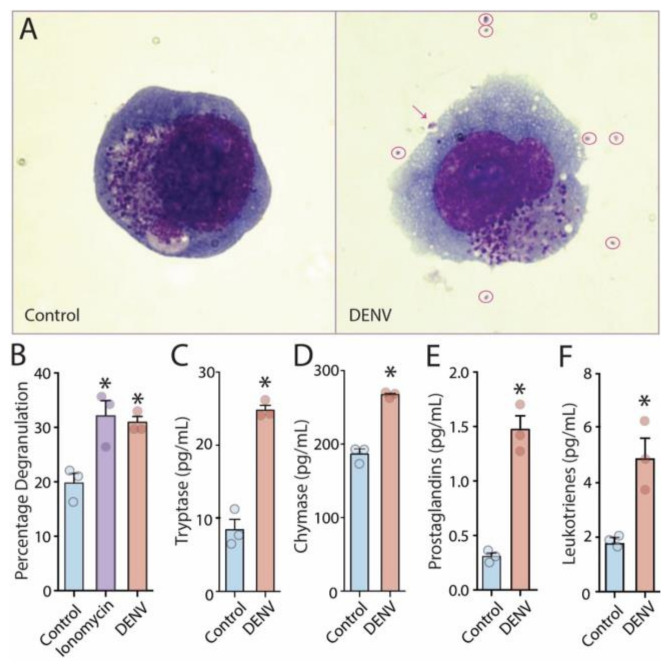
huMC activation by dengue virus (DENV) is characterized by degranulation and eiocosanoid production. (**A**) Images are representative of control-treated or DENV-activated huMCs. Control huMCs appear granulated and quiescent in comparison to DENV-activated huMCs, which degranulate. Extracellular granules are circled and an arrow points to a granule that is in the process of being released from the huMC surface. Images are representative of multiple cells observed from 3 independent experiments. (**B**) Percentage of degranulation was quantified by β-hexosaminidase assay; * indicates significant degranulation compared to control (spontaneous release) for both ionomycin and DENV stimulation (*p* < 0.05). (**C**,**D**) ELISAs confirmed release of granule-associated proteases, (**C**) tryptase and (**D**) chymase. Eicosanoid mediators, (**E**) prostaglandins and (**F**) leukotrienes are induced by DENV treatment of huMCs at 1 h post-treatment with DENV. Dots represent technical replicates. * indicates *p* < 0.05 by 1-way ANOVA (panel B) or Student’s un-paired *t*-test (panels C–F).

Since DENV largely infects only humans, we began these studies with the aim of thoroughly characterizing the vasoactive huMC products elicited by DENV activation. Importantly, we used a human clinical isolate of DENV, the Eden2 strain, which is a well-characterized low passage isolate of DENV serotype-2 [7]. We observed that huMC degranulation after exposure to DENV (Figure 1A,B) corresponded with detection of the proteases tryptase (Figure 1C) and chymase (Figure 1D) in cell culture supernatants. These proteases are understood to be granule-associated proteases that have diverse roles in endothelial activation, vasoconstriction, vascular permeability and coagulation [26]. DENV exposure also elicited release of de novo synthesized eicosanoid mediators, both prostaglandins (Figure 1E), which have a prominent role in reducing platelet aggregation and promoting vasodilation [4], and leukotrienes (Figure 1F), which induce microvascular permeability [4]. These products were significantly detected 1 h post-treatment with DENV (Figure 1E,F).

**Figure 2:**
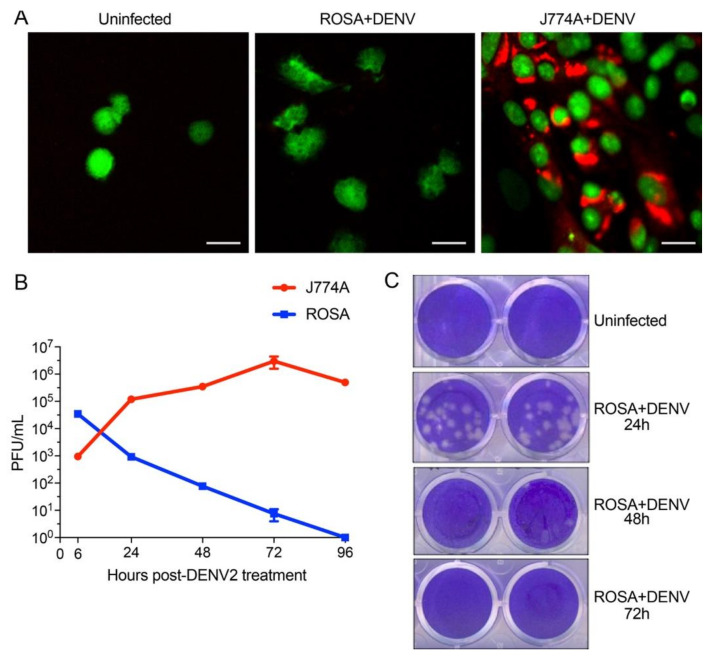
huMCs are not productively infected by DENV. (**A**) Images of control ROSA cells, DENV-exposed ROSA cells, and DENV-permissive macrophage (J774A) cells 48 h after exposure to DENV. Cells were stained for nuclear DNA (green) and DENV Env protein (red). Accumulation of Env protein was only visually apparent in the positive-control macrophage cells. (**B**) Kinetics of DENV titers in the supernatants of DENV-exposed ROSA and J774A cells, quantitated by plaque-forming assay. Error bars represent the SEM and are present at all time points, even if not visually discernable. (**C**) Images of representative plaque assay duplicates after titering of supernatants from ROSA-exposed MCs at various time points are shown. Residual input virus plaques were observed at early time points and decreased over the course of incubation. Productive replication of DENV was not observed in huMCs.

We next questioned whether DENV replicates in huMCs. First, we exposed huMCs to DENV in cell culture and, after 48 h, we fixed the cells and stained for the accumulation of DENV envelope protein (Env) antigen in the cytoplasm (Figure 2A). As a positive control, we used the macrophage cell line J774A.1 cells as infection targets, since they are known to be permissive to DENV replication [27]. Although DENV Env protein could be detected in infected macrophages, huMCs did not appear to support DENV infection since Env protein could not be detected. To support this, we also performed plaque assays on the supernatants of DENV-exposed huMCs and macrophages. DENV requires at least 12 h to complete its replication cycle, so we included a 6 h time point to quantify the input virus. Although the macrophage cell line sustained productive replication, as shown by the expansion of virus titers in the culture supernatants, huMCs showed no amplification of DENV following exposure (Figure 2B), indicating that huMCs do not efficiently support DENV replication. Indeed, the titers continued to fall over the course of 72 h (Figure 2B,C). Representative plaque assay replicates are shown in Figure 2C. These results support our previous observations with rodent MCs and indicate that, in spite of their ability to degranulate in response to DENV (Figure 1), huMCs also do not produce infectious DENV particles. 

### 3.2. Transcriptional Signatures in huMCs in Response to DENV

**Figure 3:**
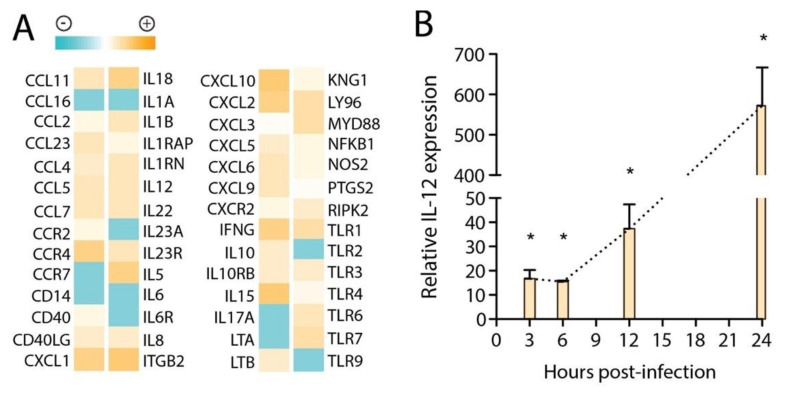
Th1-polararized transcriptional response by DENV-activated huMCs (**A**) The heat map depicts the relative expression levels of multiple genes assayed by real-time PCR array, with highest expression depicted as dark orange and lowest as dark blue. (**B**) *IL-12* expression levels in huMCs, relative to untreated huMC control levels, measured by real-time PCR after DENV exposure at an MOI of 1. * indicates a significant increase over baseline levels by 1-way ANOVA (*p* < 0.05).

To characterize the mediators that were de novo transcribed by huMCs upon exposure to DENV, we assessed the transcriptional response. For this, we compared resting huMCs to MCs treated with DENV at MOI 1 and observed broad activation of pro-inflammatory pathways, as determined by a PCR array at 24 h. This approach was used since limited information is known about the transcriptional regulation of MCs in response to DENV, specifically, or even, broadly, to viral pathogens. Genes both detected and differentially regulated between control and DENV-treated huMCs are presented in Figure 3A. Interestingly, there were several genes most strongly associated with anti-bacterial responses that were expressed at baseline in huMCs including *TLR2* and *CD14*, which were downregulated in response to DENV. As expected, anti-viral response-associated genes were upregulated, including *TLR3* and *TLR7*. *Kinnogen-1*, the precursor of the peptide bradykinin, which influences blood pressure and vascular tone [28], was also induced. Additionally, we observed a polarization towards a primarily Th1 or, to a lesser extent, Th17-inducing cytokine profile based on increased expression of certain key cytokines and inflammatory mediators, including *IFN-γ*, and reduced expression of Th2-associated *IL-6*. We also performed real-time PCR for *IL-12* along an extended time course, since this is a key Th1-promoting cytokine, which confirmed the strong induction of *IL-12* transcription by huMCs in response to DENV (Figure 3B). Cumulatively, these assessments supported a predominantly Th1-polarized and anti-viral activation profile for DENV-stimulated huMCs, with concurrent release of many vasoactive factors involved in vascular leakage, regulation of the coagulation cascade, and regulation of vascular tone. 

### 3.3. MC-Induced Endothelial Activation

**Figure 4:**
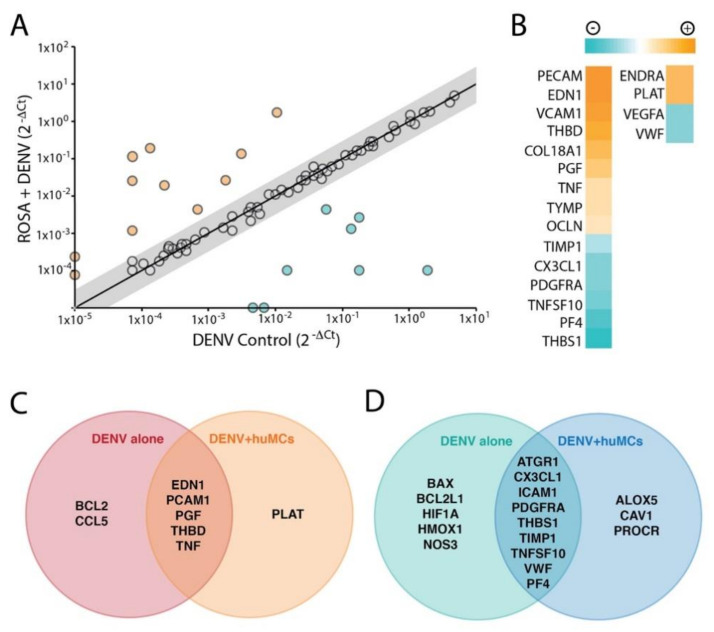
DENV-elicited MC products induce endothelial activation. (**A**) The graph shows the expression levels of unique genes over baseline expression in huMECs, 24 h after treatment with DENV alone or huMC + DENV. (**B**) A heat map of up- and down-regulated genes after treatment with supernatants from huMC + DENV, which were compared to DENV alone. (**C**,**D**) The up- and down-regulated genes are presented as Venn diagrams, representing the genes that were either similarly (**C**) upregulated or (**D**) down-regulated by DENV alone exposure and huMC products elicited by DENV (huMC + DENV) compared to control huMECs. Genes without significantly different levels of expression compared to control between DENV and huMC + DENV are located in the overlapping regions in C–D. Unique regions in the Venn diagrams that do not overlap designate genes that were uniquely expressed by only one treatment condition, whereas expression was not detected in the other treatment condition.

Due to their close physical proximity, perivascular MCs have the potential to influence endothelial cell homeostasis and activation at sites of infection and inflammation [4]. Moreover MC-proteases, chymase and tryptase can directly breakdown endothelial tight junctions [18]. To specifically explore the influence of DENV-elicited huMC products on endothelial activation, we chose to assess a broad transcriptional panel of relevant genes by PCR array and then undertook a trans-well assay to determine whether DENV-evoked MC products induced tight junction breakdown. These experiments were designed to address whether MC vasoactive products directly induce microvascular permeability by breaking inter-endothelial junctions. After exposure of huMCs to DENV at an MOI of 1 for 2 h, supernatants from DENV-activated MCs or unactivated huMC supernatants or DENV-containing media alone were transferred onto cultured huMECs. HuMEC RNA was collected 24 h after stimulation and cDNA was synthesized for PCR array analysis. We found that supernatants from DENV-treated huMCs induced huMECs to upregulate several genes that have known functions to reduce blood clotting, promote vascular leakage, and induce vasoconstriction, compared to DENV-treatment alone (Figure 4A, Table 1), and to downregulate expression of genes for products that are responsible for promoting platelet aggregation and repairing damaged cellular matrixes, compared to the virus control treatment (Figure 4B, Table 2). A number of genes were also either induced (Figure 4C) or suppressed (Figure 4D) by both DENV and huMC supernatants containing DENV compared to untreated huMCs or uniquely upregulated (Figure 4C) or downregulated (Figure 4D) compared to untreated huMCs. Thus, DENV-elicited MC products induce transcriptional changes in endothelial cells that are consistent with endothelial activation and initiate endothelial cell intrinsic responses that are associated with enhancing permeability.

### 3.4. DENV-Induced huMC Products Promote Endothelial Permeability

**Figure 5:**
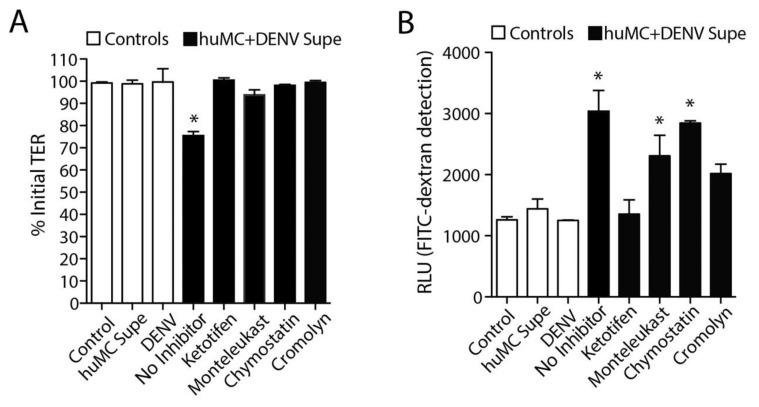
MC-stabilizing drugs block permeability of huMEC monolayers. (**A**) The TER was measured across huMEC monolayers 24 h after control treatments, treatment with the supernatants of DENV-activated MCs, or supernatants of HuMCs activated by DENV in the presence of MC modulating drugs. * Indicates a significant decrease over control, determined by 1-way ANOVA (*p* < 0.05). The average baseline TEER in control cells was approximately 220 Ω × cm^2^. (**B**) Corresponding measurements of FITC-dextran flux across huMEC monolayers were obtained 48 h after treatment. * Indicates a significant increase over control, determined by 1-way ANOVA (*p* < 0.05).

We next aimed to investigate how DENV-elicited huMC products influenced the permeability of huMEC monolayers in our coculture system. We previously used a trans-well assay to show that activation of murine MCs in response to DENV infection promoted increases in permeability of mouse endothelial cell monolayers [14] and that the MC-derived proteases chymase and tryptase were sufficient to induce permeability of endothelial cell monolayers [18]. Here, we aimed to confirm and extend upon these findings by using a trans-well culture system. Here, huMCs were activated by treating the cells with DENV for 2 h, followed by isolation of the cell culture supernatants. When products released from DENV-activated huMCs were used to treat huMECs, the TER of the huMEC monolayers was reduced, indicating increased permeability, while no significant changes were observed in the control groups, including DENV alone (Figure 5A). Increases in the monolayer permeability were also indicated by detection of increased diffusion of the large molecule FITC-dextran across the monolayer after treatment with DENV-induced MC products (Figure 5B). Again, none of the control groups (untreated, supernatant from unactivated MCs, or DENV alone) showed any signs of increased permeability or FITC-dextran penetration (Figure 5B). As we observed previously using mouse cell cultures [14], drugs that blocked huMC degranulation in response to DENV, cromolyn and ketotifen, also significantly limited both the drop in TER and the diffusion of FITC-dextran across the trans-wells that occurred without inhibitors (Figure 5A,B). The leukotriene receptor antagonist, montelukast, and the chymase inhibitor, chymostatin, also showed moderate efficacy with regards to FITC-dextran leakage (Figure 4B), but did not influence the TER measurements (Figure 5A). Thus, inhibitors of MC degranulation, leukotriene blockers, and specific inhibitors of MC proteases were effective to varying degrees in limiting breakdown of tight junctions of huMECs.

**Figure 6:**
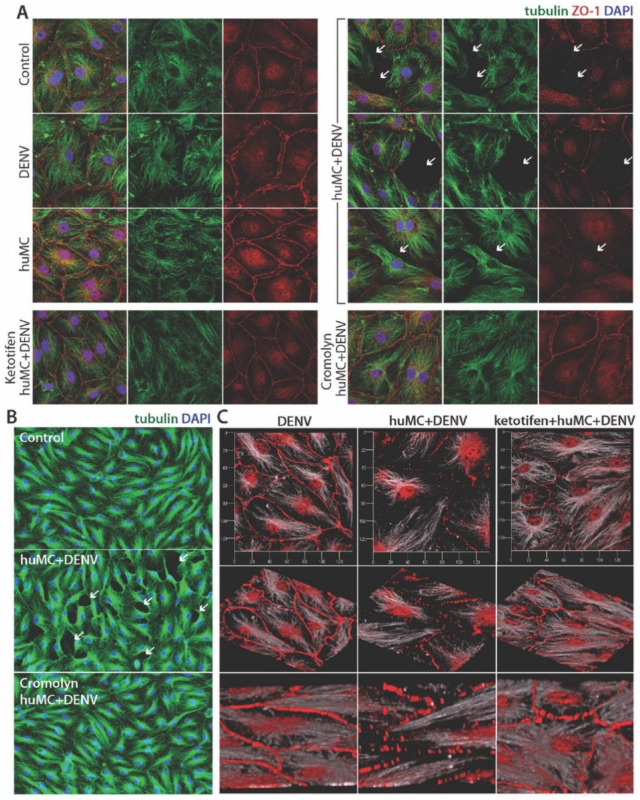
Tight junction breakdown due to DENV-induced MC products is prevented by MC stabilization. (**A**) huMEC monolayers were treated for 24 h with either media alone (control), DENV alone (3 × 10^6^ pfu/mL), media from untreated huMCs, media from DENV-treated huMCs, (3 × 10^6^ pfu/mL; MOI = 1), or DENV-treated huMCs that had been pretreated with MC-stabilizing drugs cromolyn or ketotifen. Monolayers were then imaged at 63× magnification by confocal microscopy, after staining for cellular structures including the microtubule networks (tubulin, green), the cell nucleus (DAPI, blue), and tight junctions (ZO-1, red). Regions where adjacent cells appear separated or cells appear to have lifted off of the coverslip surface are denoted with white arrows. (**B**) Widespread gap formation between cells in huMEC monolayers treated with DENV-induced MC products can be observed at lower (20× magnification), compared to untreated control groups and cromolyn-pretreated groups. (**C**) 3D-reconstructions were made from z-stacks of huMC monolayers. Here, the cell shape and tight junctions are shown through staining with ZO-1 (red) and tubulin (white). For cells exposed to huMC products after DENV treatment, punctate staining could be observed along cellular junctions, whereas, for the DENV alone control and ketotifen treated MC + DENV groups, thick and uninterrupted staining for tight junction marker ZO-1 appears at cellular intersections.

To confirm the influence of MC products on huMEC monolayer permeability in this system with a technique allowing visualization, huMECs were grown on coverslips, treated, as above, with DENV-activated MC supernatants or appropriate controls, and stained to reveal cellular junctions and cytoskeletal structures. The tight junctions between cells were stained for the junctional protein ZO-1, revealing a strong and even barrier between adjacent control huMECs, as well as huMECs after DENV treatment alone, or treatment with unstimulated MC supernatants alone (Figure 6). Supernatants from DENV-activated MCs promoted tight junction loss, based on decreased staining for ZO-1 and lifting of adjacent cells away from the coverslip that resulted in gap formation between cells. These changes were visible at both high (Figure 6A) and low magnification (Figure 6B). 3D-reconstructions of huMEC monolayers after confocal microscopy revealed punctate staining for ZO-1 and extensive loss of cell attachment sites between adjacent huMECs when cells were treated with DENV-activated huMC supernatants (Figure 6C). Consistent with our observations in the studies quantitating trans-endothelial permeability (Figure 5), tight junction integrity was restored by pre-treatment of huMCs with MC-stabilizing drugs, either cromolyn or ketotifen (Figure 6A–C). Thus, doses of virus that do not initiate endothelial permeability alone can prompt huMCs to release vasoactive factors that degrade tight junctions and allow permeability of huMEC monolayers. However, MC stabilizing drugs can limit this vascular leakage induced by DENV-activated huMCs. 

## 4. Discussion

Here, we show that DENV-mediated activation of huMCs is characterized by induction of Th1-associated cytokines, de novo production of vasoactive factors and degranulation. This contrasts the classical view that MCs are essentially Th2 polarized and polarizing cells. We also observed that the MC products that are produced immediately, within 1 h of huMC exposure to DENV, promote significant breakdown of the tight junctions between huMECs, allowing permeability in endothelial monolayers. That products contained in the supernatants of activated MCs at this short time point after exposure to DENV can induce microvascular permeability suggests that the preformed and prestored products that are released during DENV-induced degranulation are sufficient to induce vascular leakage, and that the de novo produced cytokines which take several hours to be released are not required. This 1 h time point is far shorter than the approximately 12 h window of time that DENV replication requires [29], but we also did not observe productive DENV replication in huMCs as measured by plaque assay and intracellular staining for DENV. This is consistent with our previous observations in rodent MCs, which were also not significantly infected by DENV [7]. Using this huMC line that has a highly mature phenotype is an advantage for understanding whether mature huMCs in vivo could become infected. Since MCs are only mature in tissues while their immature precursors are found in the blood [30], the responses of huMCs to DENV have been difficult to ascertain in vivo. MC-specific biomarkers found in the serum of DENV patients have primarily been used to confirm the activation of huMCs during infection [31], but this method cannot provide information on the permissiveness of the cell type to DENV infection. The lack of permissiveness of the highly mature huMC cell line ROSA, used here, to DENV replication supports that mature MCs may not be a major target of DENV infection in humans. We also observed that DENV alone at the MOI we used was sufficient to induce transcriptional activation of endothelial cells, but insufficient to lead to microvascular permeability. Together, these observations emphasize the importance of huMC detection of DENV, rather than infection by DENV, and the subsequent degranulation response of huMCs in leading to the release of vasoactive mediators.

Many experimental in vitro studies of DENV-induced vascular leakage use treatment of cells with DENV-specific antibodies as a methodological approach to generate heightened levels of infection in cell culture. This phenomenon, where cells are infected at high levels based on the uptake of virus-antibody immune complexes by Fc receptors, is called antibody-dependent enhanced infection (ADE). The rationale for using ADE is that it is the prime theory for why human DENV patients are more likely to have severe symptoms during a secondary DENV infection [32,33]. We have previously shown that DENV-specific antibodies can also enhance the degranulation responses of rodent MCs, with a mechanism based on cross-linking of the Fc receptor, and suggested an alternate method by which antibodies could enhance release of vasoactive mediators during a secondary infection [16]. Here, we chose to examine the direct (antibody-independent) responses of huMCs to DENV to be able to define the intrinsic responses that are DENV-specific and independent of the Fc Receptor signaling cascades, which would certainly modify the response. This is also a valid line of inquiry since many human patients experience severe disease during primary DENV infection [12].

In addition to the direct break down of tight junctions by MC-derived products, we found that huMECs also respond to DENV by upregulating many cytokines, chemokines and cytokine receptors associated with a Th1 polarization profile, for example IL-18 and IFN-γ. IL-18 has been shown to suppress the Th2 cytokine response and limit IgE production when combined with IL-12 [34]; although it should be noted that IL-18 alone has been reported to have the opposite effect, promoting IgE production [35]. Other interesting examples of DENV-induced cytokines/chemokines in huMCs include IL-15, which can augment T cell activation and proliferation [36], and CXCL10, which promotes T cell recruitment. CXCL10 and CCL5 were both also previously reported to be induced by DENV-treated rodent MCs [7], supporting that MC responses to DENV are broadly conserved across multiple species [7]. TLRs associated with detection of viral products, including TLR3 and TLR7, were also upregulated. Measurement of IL-12 responses over a time course of 24 h after exposure of huMCs to DENV by real-time PCR confirmed the increased transcription of this Th1-associated cytokine. These MC transcriptional activation responses clearly show a perceived viral challenge and represent the induction of a classically anti-viral response.

Endothelial cells responded to the products that were released by DENV-activated huMCs by increasing transcription of several genes that function to reduce blood clotting and by downregulating genes whose products are responsible for promoting platelet aggregation and repairing damaged extracellular matrixes. For example, plasminogen activator (*PLAT*) encodes a protease that converts plasminogen to plasmin to reduce clotting and dissolve fibrin [37]. Endothelin-1 (*EDN1*) and Endothelin receptor type-A (*ENDRA*) were also found to be upregulated and EDN1 previously was shown to contribute to the pathogenesis of hemorrhagic shock resulting from gastric mucosal damage, analogous to gastric ulcer [38]. Chymase, which we observed is released as a component of granules during the degranulation response, also can convert Endothelin-1 to its active form [39], indicating the potential of combined activity by MC and endothelial cell derived mediators. In contrast to DENV treatment of endothelial cells, DENV-induced MC-products also reduced expression of the von Willebrand factor (*VWF*), which is essential for platelet aggregation sites of vascular damage [40]. The dramatic permeability and endothelial activation we observed did not occur with physiological doses of DENV alone, but only in the presence of DENV and the vasoactive products that are released by MCs treated with DENV. This supports that the mediators produced by MCs, including their granule-associated products and eicosanoids, are important for degrading tight junctions and inducing endothelial permeability. It is also possible that vascular leakage in vivo could involve the combined effects of MC products that promote endothelial activation and permeability, along with those products that reduce clotting and limit the coagulation cascade, such as heparin (released during MC degranulation) and VWF (produced by MC-activated endothelial cells) [4].

Since many MCs products are known to have synergistic effects on the vasculature, we anticipate that multiple MC-derived products are functioning in combination to induce vascular leakage. In support of this, drugs inhibiting either the production or activity of several individual vasoactive mediators (e.g., proteases inhibitors, leukotriene receptor antagonists) or targeting a combination of vasoactive mediators (e.g., MC stabilizers) were all capable of reducing huMC-induced endothelial permeability and promoting tight junction integrity. MC stabilizing drugs were the most effective class of drug tested here in reducing endothelial permeability, and one of these drugs, ketotifen, was shown to effectively reduce inflammation in a mouse model of dengue [15] and is currently being tested for efficacy in reducing DENV vascular leakage in a human clinical trial. The findings of this study show that the products released by huMC in response to DENV have the potential to promote endothelial permeability, but that targeted approaches for blocking the activity of MCs on vascular endothelium are promising candidates for treating the vascular leakage that occurs during human DENV infection.

## Figures and Tables

**Table 1:** Vascular functions of products with increased expression in HuMEC cells in response to DENV-activated huMCs.

Vasoactive Factor		Major Described Biological Functions
**PECAM (CD31)**	Platelet endothelial cell adhesion molecule	Promotes leukocyte transmigration
**EDN1**	Endothelin-1	Vasoconstriction
ENDRA *	Endothelin receptor type A	The receptor for Endothelin-1, Vasoconstriction
PLAT *	Plasminogen activator	A protease that converts plasminogen to plasmin, which dissolves fibrin and reduces clotting
**THBD**	Thrombomodulin	Thrombin receptor that promotes degradation of clotting factors
**COL19A1**	Collagen, type XIX, alpha 1	Extracellular matrix protein
**PGF**	Placental growth factor	A member of the VEGF family with diverse roles in angiogenesis and vascular function
**TNF**	Tumor Necrosis Factor	A cytokine with broad roles in host defense. Also promotes processes such as fever, cell death, and vascular leakage
**TYMP (ECGF1)**	Thymidine phosphorylase	Promotes angiogenesis
**OCLN**	Occludin	Tight junction protein that regulates paracellular permeability

* Produced in HuMECs treated with DENV-elicited MC products but not detected in DENV treated HuMECs.

**Table 2:** Vascular functions of products with decreased expression in HuMEC cells in response to DENV-activated huMCs.

Vasoactive Factor		Major Described Biological Functions
**TIMP1**	TMP metallopeptidase inhibitor	Inhibitor of matrix metalloproteinases, which degrade extracellular matrix
**CX3CL1 (Fractalkine)**	CX3CL1 chemokine (C-X3-C motif) ligand 1	Leukocyte migration (primarily T cells and monocytes)
**PDGFRA**	Platelet-derived growth factor receptor, alpha polypeptide	Activation promotes angiogenesis
**TNFSF10**	Tumor necrosis factor (ligand) superfamily, member 10	Pro-apoptotic protein in stressed but not normal cells
**PF4**	Platelet factor 4	A member of the CXC chemokine family that promotes platelet aggregation
**THBS1**	Thrombospondin 1	An adhesive glycoprotein that promotes cell–cell interactions
VEGFA **	Vascular endothelial growth factor A	Promotes vascular permeability and angiogenesis
VWF **	von Willebrand factor	Performs a critical function in coagulation

** Produced in HuMECs treated with DENV but not in HuMECs treated with DENV-elicited MC products.
